# Gene Expression of *Mycobacterium tuberculosis* Putative Transcription Factors *whiB1-7* in Redox Environments

**DOI:** 10.1371/journal.pone.0037516

**Published:** 2012-07-19

**Authors:** Christer Larsson, Brian Luna, Nicole C. Ammerman, Mamoudou Maiga, Nisheeth Agarwal, William R. Bishai

**Affiliations:** 1 Center for Tuberculosis Research, Johns Hopkins University School of Medicine, Baltimore, Maryland, United States of America; 2 Vaccine and Infectious Disease Research Center Translational Health Science and Technology Institute, Gurgaon (Haryana), India; 3 KwaZulu-Natal Research Institute for Tuberculosis and HIV, Durban, South Africa; 4 Howard Hughes Medical Institute, Chevy Chase, Maryland, United States of America; University of Hyderabad, India

## Abstract

The seven WhiB proteins of *Mycobacterium tuberculosis* (*M.tb*) are widely believed to be redox-sensing transcription factors due to their binding of iron-sulfur clusters and similarities to DNA binding proteins. Here, we explored the nature of this hypothesized relationship. We exposed *M.tb* to physiologic conditions such as gradual hypoxia, nitric oxide (NO), cyclic AMP and *in vivo* conditions, and measured transcription of the *whiB* genes. We found *whiB3* to be induced both by hypoxia and NO, *whiB7* to be induced in macrophage-like cells, and *whiB4* to be induced in mouse lung. Cyclic AMP induced *whiB1,−2, −4, −6* and *−7*. Our data indicate that the *M.tb whiB* genes are induced independently by various stimuli which may add versatility to their suggested redox-sensing properties.

## Introduction

*Mycobacterium tuberculosis* (*M.tb*) is one of the world’s most successful pathogens. Approximately one-third of humanity is infected, but only 5–10% of this population develops active disease, which in 2010 accounted for 8.8 million cases of tuberculosis (TB) and 1.45 million deaths [1]. The remaining 90–95% either clears the infection or carries a latent or quiescent infection remaining for extensive time, often for life. This requires an extraordinary adaptation to the various and often extreme environments the bacteria meet in the host such as nitric oxide (NO) and low pH in the phagosome, or months or years in hypoxic or even anaerobic compartments such as caseous granulomas. In this state of latency bacteria still have to be able to respond to stimuli to become active again when conditions to do so are favorable.

Availability of oxygen is fundamental for *M.tb* metabolism and may be important for the entering into latency and triggering reactivation [2]. The most well-studied oxygen-sensing gene regulator in *M.tb* is the DosR/DevR two-component signaling system [3], but there are likely other systems also responding to redox changes. One group of proteins that has been suggested is the WhiB family. The WhiB-like proteins are unique to *Actinomycetes*. They are small (75–130 amino acids) with 4 conserved cysteine residues typical for metal-coordinating DNA-binding proteins, and these proteins also have a conserved aspartate and a helix-turn-helix-like motif [4]. The 4 cysteines bind a [4Fe-4S] cluster which changes the conformation of the protein in a redox-dependent manner [5], [6]. Together with the DNA binding properties of helix-turn-helix-like structures, the WhiB proteins are hypothesized to be transcription factors binding to or releasing DNA depending on redox potential in a manner similar to that of *E. coli* FNR [7], [8].

The first protein in this family (WhiB) was discovered in *Streptomyces coelicolor* and found to be essential for sporulation [9]. The *M.tb* genome contains seven orthologs named *whiB1-7,* of which only *whiB2* is postulated to be an essential gene [10]. Apart from the conserved motifs described above, the sequences of the seven genes are not very similar, indicating different functional properties. Further, the mycobacteriophage TM4 carries a *whiB* homolog similar to *whiB2* binding to the *whiB2* promoter region and inhibiting its synthesis [11].

In a previous study, Geiman et al. found that transcription of *M.tb whiB2* was strongly down-regulated in late stationary phase compared to early log phase, while, in contrast, transcription of *whiB3* was more than 20-fold induced at late stationary phase, and more than 10-fold induced at low pH [12]. *whiB6* seemed to be more of a general stress responder, being induced by various *in vitro* stresses such as sodium dodecyl sulfonate, ethanol, cumene hydroperoxide, diamide and incubation at 42°C. *whiB7* expression has been reported to be induced by antibiotics inhibiting protein synthesis such as streptomycin and kanamycin, heat shock, iron starvation and palmitic acid [12], [13], [14]. Interestingly, both Mycobacterial and Streptomyces *whiB7* null mutants are hypersensitive to antibiotics, suggesting a role for WhiB7 in antibiotic tolerance [13].

Our objective was to mimic various physiological conditions that *M.tb* are likely to encounter *in vivo,* with emphasis on prolonged exposure (hours) rather than shock response (minutes), and to analyze the *whiB* response to these conditions. We studied redox environments, such as the slow, self-generated oxygen depletion of the Wayne model, mimicking the progressive hypoxia during granuloma formation, and incubation with DETA-NO, modeling the phagosomal NO burst. We also assessed *whiB1-7* expression in macrophage-like cells *in vitro* and in caseous, hypoxic granulomas *in vivo* [15], [16]. Finally, we evaluated the importance of cyclic AMP, a signaling molecule important in both prokaryotic and eukaryotic gene regulation, on *whiB* expression [17]. Our data indicate that the *M.tb whiB* genes respond to several different stimuli in a non-coordinate manner, suggesting unique functions of the seven genes and their cognate protein products.

## Results

**Figure 1:**
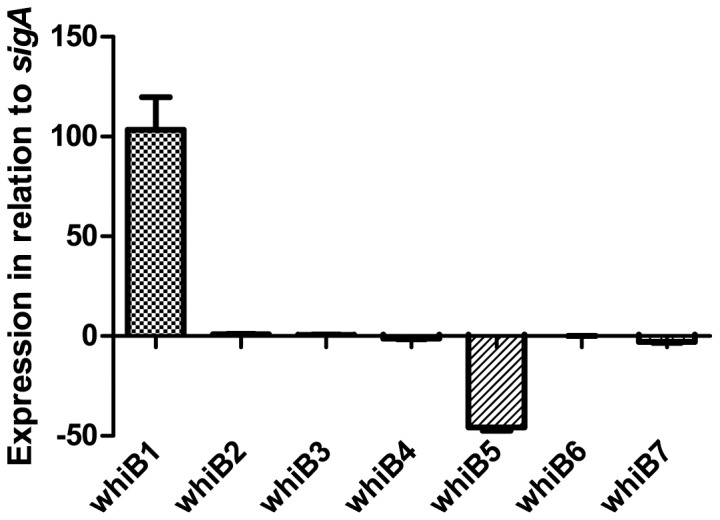
Relative abundance of *whiB1-7* transcripts. mRNA levels of the seven *M.tb whiB* genes at mid-logarithmic growth in 7H9 presented as fold change in relation to *sigA* (set to 0). *WhiB1* was highly expressed and *whiB5* was under-expressed compared to the other *whiB* genes, which exhibited an expression level very close to that of *sigA*.

The WhiB proteins are believed to be redox sensing transcription factors. To determine the conditions in which WhiB1-7 are utilized, *M.tb* were exposed to various conditions likely to occur in physiological environments with emphasize on redox change. The *whiB* transcript abundance was fairly consistent and similar to the expression level of housekeeping gene *sigA* throughout the *whiB* family with the exception of *whiB1* being about 100-fold higher and *whiB5* being about 50-fold lower than *sigA* transcripts (and the rest of the *whiB* gene family) when grown in 7H9 broth under aerobic conditions (Figure 1). We found *sigA* to be most suitable as a housekeeping gene in our experiment since its expression was close to the levels of the majority of *whiB* genes (Figure 1), and it has been previously used in other similar studies [12]. The 16S rRNA sequence was considered but was too highly expressed (Ct ∼6) to provide accurate comparisons (data not shown). Although there is evidence of interactions between SigA and WhiB3 on a protein level [18], there was no suggestion of interaction on a transcriptional level in our experimental system. None of the target transcripts were ever undetectable, suggesting a fairly robust basal expression level of all *whiB* genes in *M.tb*.

**Figure 2:**
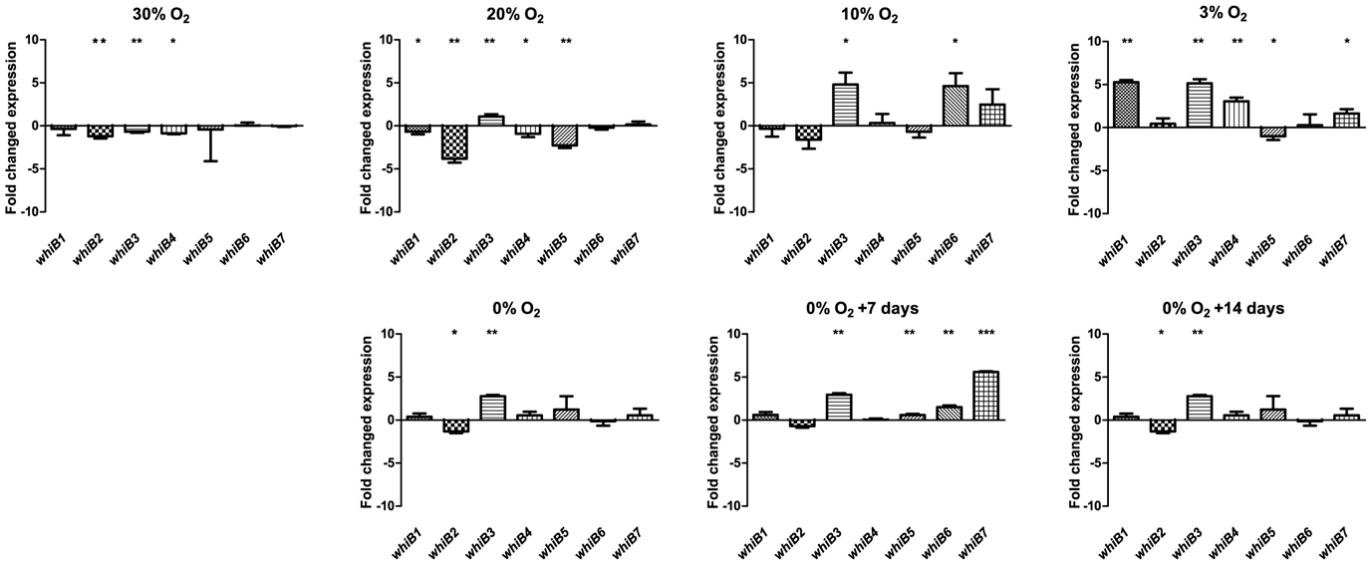
*M.tb whiB1-7* expression in a self-generated oxygen depletion (Wayne) model. Data are presented as fold-change compared to fully aerated (100% air saturation) cultures denoted as 0 on the y-axis. All genes were expressed in all conditions. *whiB3* showed a consistent trend of upregulation starting at about 20% a.s. *whiB2* showed a trend of slightly lower expression in low oxygen conditions although not reaching statistical significance at 10% and 0%+7days (p = 0.09 and 0.06 respectively) and with an apparent, non-significant upregulation at 3%.

The level of oxygen has a great effect on *M.tb* cell division, metabolism and gene expression as reviewed in [2]. The Wayne model, in which bacteria are grown under slow stirring in sealed flasks, provides time for adaptation to the hypoxia generated by the bacteria’s own respiration [19]. By using a fine needle to penetrate the sealing rubber septae, the O_2_ content was measured with an OXY MICRO O_2_ meter and cultures were harvested at various air saturations (Figure S1). The 0% air saturation was harvested on the day of methylene blue color fading and remaining cultures were harvested 1 and 2 weeks after color change. The expression of *whiB1-7* was surprisingly robust throughout the transition from aerobic to anaerobic conditions (Figure 2). Most noteworthy was *whiB3* which demonstrated upregulation at about 20% air saturation which increased and remained elevated into the late anaerobic phase of the experiment. *whiB2* showed a slight downregulation at the early microaerobic phase, and *whiB1*, *6* and *7* were to a lesser extent upregulated at 3%, 10% and 0%+7 days, respectively (Figure 2). Oxidative stress, induced by a 1-hour incubation in 1 mM H_2_O_2_ did not affect the expression of any of the *whiB* genes, nor did DNA damage induced by UV irradiation, indicating that whiB proteins may not play a critical role in DNA damage response or DNA repair mechanisms (data not shown).

**Figure 3:**
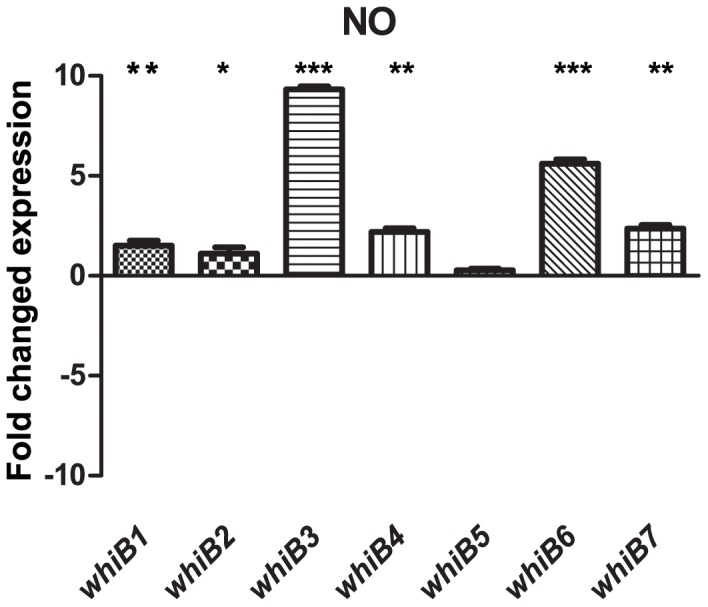
Effect of nitric oxide on *whiB1-7* expression. *M.tb* CDC1551 was grown for 12 h in the presence of 500 µM DETA-NO. *whiB3, whiB6* and to a lesser extent, *whiB7* were upregulated.

NO is released by macrophages as part of the innate immune response and is a redox active, oxidative compound. To assess the impact of NO exposure on *whiB1-7* expression, bacteria were treated with the compound DETA-NO, which causes a release of NO into the media upon degradation. The 500 µM concentration and 12 h time point used was chosen based on the thorough work by Voskuil et al. where they tested various concentrations of DETA-NO, its dissociation kinetics over time, and its effect on cell division and NO induction of the *M.tb* dormancy program [20]. Since the NO is produced by the decay of DETA-NO but also consumed in various reactions by its high reactivity, the time point was chosen to avoid depletion of DETA-NO in the cultures. *whiB3* was induced 9-fold and *whiB6* 6-fold, while *whiB7* showed a moderate 2-fold induction compared to the untreated controls (Figure 3).

**Figure 4:**
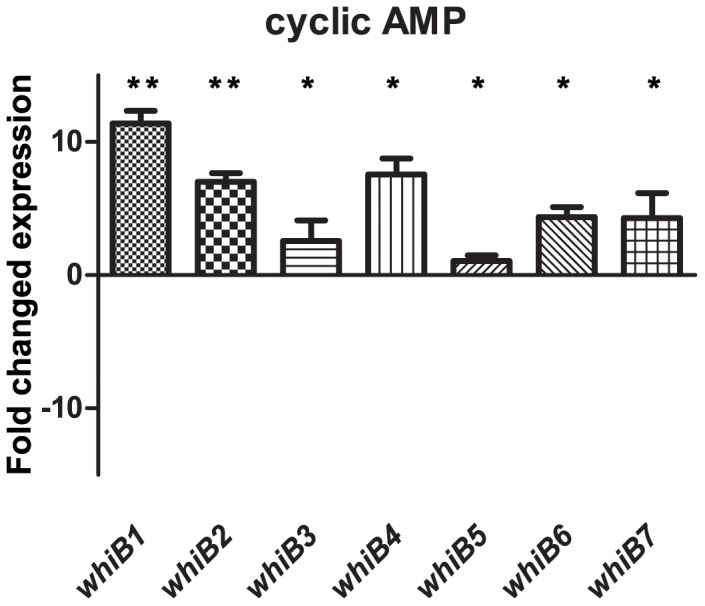
Effect of db-cAMP on *whiB1-7* expression. *M.tb* CDC1551 was grown 24h in the presence of 500 µM db-cAMP. *whiB1* and to a lesser extent *−2* and *−4* were up-regulated.

Agarwal et. al found that *M.tb whiB1* expression is regulated by cyclic AMP through the cAMP receptor protein (Crp) [21]. To assess the role of cAMP regulation for all *M.tb whiB* genes, cultures were incubated with 500 µM di-butyryl cAMP, a cell membrane permeable analogue (in contrast to cAMP). This concentration was chosen since it was the lowest concentration that gave a clear Crp binding to the *whiB1* promoter in [21]. We found all *whiB* genes to be induced by cAMP, although the upregulation of *whiB5* was only 1-fold. *whiB1*, *whiB4* and *whiB2* were most affected with 11-, 8- and 7-fold upregulation, respectively (Figure 4).

**Figure 5:**
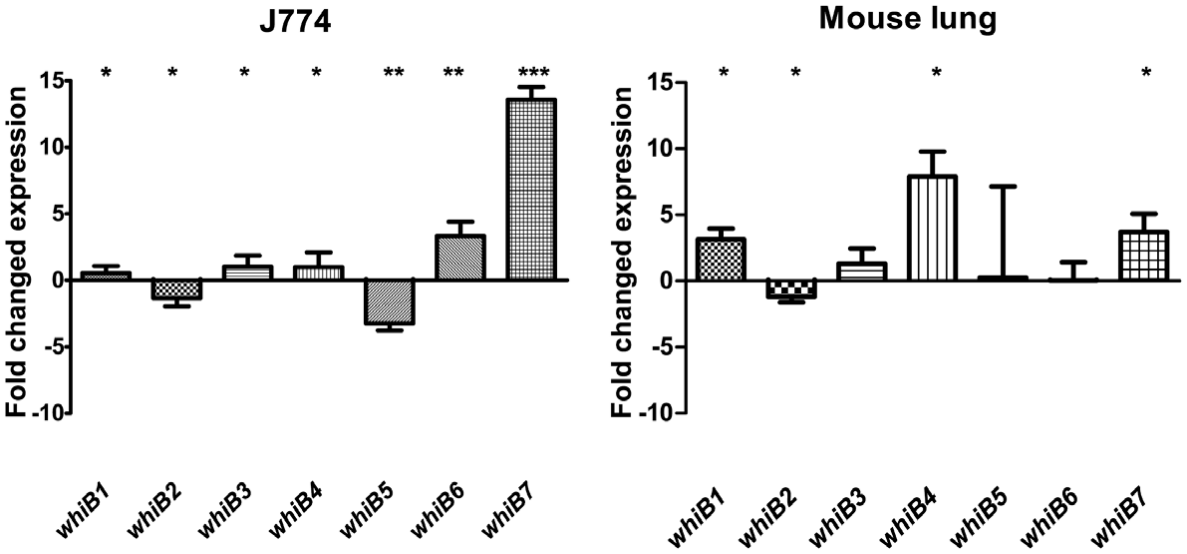
*whiB1-7* expression in J774 macrophage-like cells and lungs of C3HeB/FeJ mice. Expression levels compared to aerobic *in vitro* 7H9 growth. Only *whiB6* and *whiB7* were upregulated during J774 cell infection whereas *whiB1, −4* and *−7* were upregulated in the lungs of C3HeB/FeJ mice (5 weeks post-infection).

Two *in vivo* conditions were studied in this experiment: infection of the murine macrophage-like cell line J774 and an aerosol infection of C3HeB/FeJ mice. *whiB7* was induced 14-fold in the J774 cells, which was the highest magnitude of induction observed under any condition tested. *whiB6* was 3-fold induced, and *whiB5*, generally being very stably expressed, showed a 3-fold lower expression compared to in vitro growth (Figure 5). The most induced gene in the mouse experiment was *whiB4* with an 8-fold induction compared to in vitro growth. *whiB1* and *whiB7* were 3- and 4-fold induced, respectively (Figure 5).

To assess cross-regulation of *whiB* expression, all genes were cloned and overexpressed under an inducible acetamide promoter [22], [23]. Interestingly, the expression level varied conditionally with *whiB5* and *whiB7* being massively overexpressed (2287 and 2777 fold respectively), *whiB3* being 74-fold overexpressed down to *whiB2* with a modest 5-fold and *whiB1, −4* and *−6* not showing any overexpression at all (data not shown). When comparing expression levels of the other 6 genes to the pSCW38 vector control, none of the genes overexpressed affected the expression levels of any of the other *whiB* genes. This suggests that there were no regulatory interactions between the *whiB* genes that we were able to overexpress and other genes in the family, and that the different members of the WhiB class of proteins in *M.tb* probably have unique functions.

## Discussion

Several of the *whiB* genes can be induced 100-fold by chemical and physiological stresses and antibiotics [12] [14]. Our focus in this project was to model physiological conditions the bacteria are likely to encounter *in vivo* to find under which conditions these genes are transcribed and utilized. Overall, we found a fairly robust constitutive expression level with the highest induction being the 14-fold induction of *whiB7* in J774 cell infection.

If WhiB proteins bind and release DNA in a redox-dependent manner, one might assume that the proteins are omnipresent and subject to very little regulation on transcriptional level. This seems to hold true for some of them, e.g. *whiB5* which never differed more than 3-fold from any condition tested. On the other hand, in experiments by Geiman et al. and Burian et al., *whiB7* was upregulated 80-fold upon treatment with antibiotics binding to the 30S ribosomal subunit such as kanamycin and streptomycin [12], and over 1000-fold by exposure to erythromycin [14]. Clearly, *whiB* expression, at least for some of the genes, can be strongly induced under certain conditions, and their regulation seems to be independent and unique. This suggests they all have different functions and/or are utilized in different conditions.

Bai et al. found the promoter binding sequence of Crp to be C/TGTGANNNNNNTCACG/A [24]. By searching the 5′UTRs of CDC1551 genome using the on-line software Tuberculist (http://genolist.pasteur.fr/TubercuList/) we found that the Crp binding sequence in the 5′ UTR of *whiB1* had one mismatch to the sequence reported by Bai et al. whereas the binding sequences of *whiB2*, *3, 4* and *6* had 2 mismatches. This correlates to our expression data with *whiB1* being 11-fold upregulated by db-cAMP and *whiB2* and *whiB4* being less, 7- and 8-fold increased, respectively (Figure 4). No Crp binding sites were found in the *whiB5* or *whiB7* promoter regions and *whiB5* expression was not induced by db-cAMP treatment. A minor *whiB7* induction was seen despite lack of a Crp binding site but this may also be due to secondary effects. It is not unlikely that *whiB7* is regulated in part by another Crp-dependent protein.

Rybniker et al. found the mycobacteriophage TM4 WhiB to be able to regulate the expression of host *whiB2* [11]. To assess cross-regulation of the *M.tb* WhiB proteins, the genes were cloned into the integrating plasmid pSCW35 and expression induced with acetamide. Gene expression was compared to that of bacteria also treated with acetamide but carrying the empty vector plasmid pSCW38 (Data not shown). *whiB1,4* and *−6* could not be overexpressed and *whiB2* showed only 5-fold overexpression. However, *whiB3, −5* and *−7* showed 74-, 2287- and 2777-fold expression respectively, but none of the other *whiB* genes were repressed or induced by the treatment (Data not shown). Since we could only over-express half of the genes, we could not examine if *whiB1, −4* and *−6* can regulate expression of other *whiB genes,* but we can conclude that overexpression of *whiB3, −5* and *−7* do not. Since *whiB2* was only moderately overexpressed, it would be inappropriate to draw any drastic conclusions, but none of the other *whiB* genes were affected by the 5-fold increase in *whiB2*. Although inconclusive, we see no evidence of cross-talk between the whiB genes of *M.tb* (Data not shown). Another expression strategy, e.g. from an episomal plasmid or using another promoter may be needed for *whiB1, −4* and *−6* expression. These negative data indicate that at least *whiB3*, *−5,* and *−7* do not regulate expression of other *whiBs*. The regulation of *whiB2* by WhiBTM4, a phage encoded protein similar to WhiB2, may indicate WhiB2 auto-regulation similar to what is suggested for *whiB1* transcription where WhiB1 is believed to repress its own expression [11], [25]. The negative results indicate lack of *whiB* family cross-regulation, at least for *whiB3*, *−5,* and *−7*; suggesting independent function of these transcription factors.

In the Wayne hypoxia experiment, *whiB2* shows a trend of slightly decreased expression at low O_2_ being statistically significant at 30%, 20%, 0% and 0%+14 days and getting close to but not reaching statistical significance at 10% and 0%+7 days (p = 0.09 and 0.06 respectively) The apparent upregulation at 3% is not significant compared to control expression. The biological significance of this small downregulation is difficult to anticipate, but a balanced level of WhiB2 is important for correct septation and cell division [26]. Sherrid et al. found *whiB2* as one of 103 genes to be induced in a genome-wide microarray experiment studying reaeration response when cultures with a defined-O_2_ hypoxia were quickly reaerated to atmospheric oxygen level for 6 and 12 hours [27]. WhiB2 is the *M.tb* homologue of the well-studied WhmD of *M. smegmatis*, an essential regulator of cell septation and division [10], [26]. It is possible that WhiB2 has a role in the reactivation of *M.tb* from a latent state, but a more moderate interpretation of the data would be that it is slightly higher expressed in favorable growth conditions. However, in the experiments performed by Raghunand et al., the authors concluded that the intracellular concentration of WhiB2 was critical for correct septation [26]. *whiB3* shows a 2-fold upregulation when oxygen has decreased to 20% air saturation and is thereafter expressed 3-5-fold higher than at 100% air saturation (Figure 2). NO is produced by macrophages upon activation by cytokines or microbial products through the inducible nitric oxide synthase pathway and is a highly reactive compound killing a wide array of microorganisms. Upon NO synthesis in mammalian cells, it reacts with FeS containing enzymes inhibiting their activity [28]. Crack et al. showed that WhiB1 and the related *S. coelicolor* WhiD react extremely fast with NO, 10^4^ times faster than with O_2_ [29]. The importance of NO in host defense and the biochemistry of NO and FeS containing proteins led us to investigate the effect of NO on the *whiB* expression profile. We found upregulation of *whiB3* (9-fold) and *whiB6* (6-fold) in response to this stimulus. Interestingly, *whiB3* was upregulated by both low O_2_ and NO, both molecules with redox activity. Singh et al. showed that WhiB3 retains intracellular redox homeostasis by functioning as a metabolic switch, shifting the use of propionate in biochemical anabolism into phthiocerol dimycocerosates (PDIM), triacylglycerol (TAG), poly- and diacyltrehalose, and sulfolipids (SL-1) functioning as a reductive sink. TAG is induced via the DosR/S/T dormancy system, and the findings of Singh et al. demonstrates a link between WhiB3 and the Dos system, although WhiB3 does not seem to be a part of the DosR/S/T regulon directly [30], [31], [32]. In EMSA experiments, Singh et al. also found that WhiB3 4Fe-4S bound promoter DNA in a concentration rather than redox dependent manner. A challenge for these experiments was the low sequence specificity of WhiB3 with high binding to non-specific DNA [30]. Our data suggest *whiB3* to be important in both O_2_ and NO redox conditions and supports its function as a redox regulator. Still, it is important to remember that the intracellular O_2_ and redox potential inside the cell isn’t necessarily the same as in the external environment. When harvesting color-less, hypoxic Wayne cultures, the *M.tb* cell pellet is still bright blue from methylene blue in its oxidized form (data not shown). Before the oxygen depletion experiment, we hypothesized that the seven WhiB proteins may all be important at different oxygen concentrations. This was not the case in our experimental system, since the expression of most of them are fairly unaffected throughout the gradually increasing hypoxia although also *whiB4*, *−6* and *−7* are induced in microaerobic conditions but with no clear pattern.

The two *in vivo* conditions studied were infection of the J774 macrophage-like cell and C3HeB/FeJ mice. This mouse strain forms large necrotic lesions in the lungs upon aerosol infection [33]. *whiB7* was upregulated in both J774 cells (14-fold) and mouse lung (4-fold) and *whiB4* was 6-fold upregulated in C3HeB/FeJ mice but only 1-fold upregulated in J774 cells (Figure 5). We anticipated more similarities between the two in vivo experiments, but in the cell culture experiment, all *M.tb* are associated with a monoculture of macrophage-like cells, while a C3HeB/FeJ granuloma is a complex environment with not only macrophages, but dominated by neutrophil infiltrates and with necrotic areas containing large amounts of extracellular bacteria [34]. The bacteria from mice represent an average of bacteria from various compartments with both intra- and extracellular bacteria, bacteria from different oxygen concentrations and different exposure to immune response [15]. The in vitro parts of this study attempts to simplify and isolate single stimuli bacteria are likely to encounter to better understand the parts of the complex environment *M.tb* face during an infection.

In cell culture experiments studying intracellular bacteria, it is a common practice to kill extracellular bacteria with an antibiotic such as gentamicin. However, in experiments by Geiman et al. and Burian et al. *whiB* genes, especially *whiB7*, were massively induced by antibiotics [12], [14]. Therefore we decided to grow cells without antibiotics and only wash off extracellular bacteria with DMEM rather than kill them with antibiotics. Although not inducing transcriptional changes due to antibiotics, this procedure is less effective than gentamicin treatment in eliminating extracellular bacteria and some of the bacteria in the experiment may have been attached to cells or undergoing phagocytosis rather than residing intracellularly inside a vacuole. Surprisingly, only a negligible upregulation of *whiB3* was observed in both J774 cells and mouse lung contrasting to the observations by Banaiee et al. who observed a 10+ fold induction of *whiB3* in bone marrow macrophages and even higher in mouse lung [35]. Although surprising, the difference may be explained by different strains of bacteria, cells, mice or different methods of generating cDNA.

None of the conditions tested in this study had a major effect on *whiB5.* It only showed a 2-fold decrease at 20% air saturation and in J774 cells (Figures 2 and 5). It is likely that *whiB5* performs its mode of action solely on a protein level or, alternatively, is induced by very specific conditions not tested here. The highest induction seen in this study was the 14-fold induction of *whiB7* in J774 cell infection. Still, we see no signs of genes having paralleling transcription patterns, indicating independent regulation mechanisms for each of the seven genes with all of them, perhaps except for *whiB5*, being inducible by at least one condition. We believe the larger part of the regulatory role is on the protein level, which is supported by the fairly constitutive expression and the proposed protein conformation changes mediated by the redox sensitive [4Fe-4S] cluster [6], with some synergy with other regulatory networks, e.g. induction by cAMP signaling via the Crp binding domains in the 5′ UTR of *whiB1*, *−2* and *−4* (Figure 4). We believe the seven WhiB proteins, with their conserved FeS-binding domains but fairly low similarity apart from that, are all redox sensing but induced or repressed by different stimuli. In this current work, we demonstrate independent induction of the seven genes. Burian et. al recently showed that the reducing agent dithiothreitol (DTT) is synergistic in antibiotic mediated *whiB7* induction [14]. The 100-fold and even 1000-fold inductions seen in stress- and shock experiments compared to the clear but moderate difference in expression seen in our experiments rather aimed to mimic physiological conditions, suggest at least some of the WhiB proteins, such as *whiB6* and −*7* have an important role in stress response [12], [14]. Different members of the WhiB family probably also bind different promoter sequences and work is ongoing in our lab to characterize promoter binding in normal and low oxygen conditions. This set of experiments provides new insights in which conditions induce *whiB* transcription. To definitively determine their function, future work should focus on constructing mutants of *whiB* genes and analyzing these mutants in the conditions in which they are postulated to be important. *whiB1* and *−2* are considered to be essential, but there are mutants of *whiB3*,*−6* and *−7* of which we are currently working on a *whiB6* knockout and similar work is ongoing on *whiB3* and *−7* in other labs [13], [25], [26], [36], [37]. *whiB4* and *−5* are very little studied and have yet to be knocked out of an *M.tb* genome.

A regulatory system responding to both external stimuli on the expression level, perhaps influenced by Crp, and redox on a protein conformation level would provide a very versatile regulatory machinery for *M.tb* adaptation and understanding it will be an important step to understand mycobacterial latency and direct antibiotic therapy to key pathways in the future.

## Materials and Methods

### *M. tb* Growth Conditions

Unless otherwise stated, *M.tb* CDC1551 were grown in 7H9 broth (Becton Dickinson, Sparks, MD) supplemented with 10% OADC (oleic acid, albumin, dextrose, and catalase) (Becton Dickinson, Sparks, MD), 0.5% v/v glycerol and 0.05% v/v Tween 80 (Sigma Aldrich, St. Louis, MO) at 37°C with agitation. Cultures were grown in air atmosphere unless otherwise specified.

### Gradual Hypoxia (Wayne) Model

Gradual hypoxia was achieved using the bacteria’s own respiration, as described by Wayne and Hayes [19]. Briefly, *M.tb* CDC1551 were grown in Dubos’ medium in sealed tubes or flasks with slow stirring. Small samples were taken regularly with a thin needle through a rubber septum without letting air entering the vessels. Oxygen (% air saturation) was measured using a fiber optic oxygen microsensor (OXY MICRO, World Precision Instruments, Sarasota, FL) according to the manufacturer’s instructions. Bacteria grown to OD_600_ 0.2 in Dubos’ broth in well-aerated 1L roller bottles were used as the 100% oxygen control.

### Nitric Oxide Treatment

*M.tb* CDC1551 was grown in 7H9 supplemented with OADC (Becton Dickinson, Franklin Lakes, NJ) to an OD_600_ of 0.5. Cultures were then equally divided, with one half serving as the negative control and the other half being treated with 500 µM DETA/NO (Sigma Aldrich, St. Louis, MO) for 12 hours.

### db-cAMP Treatment

*M.tb* CDC1551 was grown in 7H9 supplemented with OADC to OD_600_ of 1. N6,2′-O-dibutyryladenosine-3′,5′-cyclic monophosphate (db-cAMP) (Axxora, LLC, San Diego, CA) was added to a final concentration of 500 µM. No db-cAMP was added to control cultures. Bacteria were harvested 24 hours after addition of db-cAMP.

### Macrophage-like Cell Infection

J774 mouse monocytic cells (J774.2, Sigma Aldrich, St. Louis, MO) were grown in DMEM supplemented with 10% newborn calf serum without antibiotics to avoid interference with gene expression. Cells were maintained at 37°C at 5% CO_2_. Cells were infected with *M.tb* CDC1551 at a multiplicity of infection of 1∶5 and incubated 24 h. Extracellular bacteria were washed away with warm medium, and infected cells were incubated 2 days before being harvested and frozen in Trizol (Life Technologies, Grand Island, NY), at −80°C.

### C3HeB/FeJ Mouse Infection

Approximately 6 week-old female C3HeB/FeJ mice were purchased from The Jackson Laboratory (Bar Harbor, ME, USA). Bacterial cultures were appropriately diluted in media to achieve the desired inoculum. Aerosol infections were performed with the Glas-col Inhalation Exposure System (Glas-Col, Terre Haute, IN), per the manufacturer’s instructions. Three mice were sacrificed the following day to determine the day 1 lung implantation. At 5 weeks post infection, mice were euthanized using isoflurane and immediately dissected. Lungs were dissected and homogenized in phosphate buffered saline. The lung homogenates were then serially diluted and plated in duplicate on selective 7H11 agar plates (Becton-Dickinson, Franklin Lakes, NJ). Plates were incubated for 3 weeks at 37°C and colonies were counted. (The day-1 count was 3.67 log_10_CFU/mouse). Lungs were cut into 2–3 mm pieces and put in RNA*later* solution (Life Technologies, Grand Island, NY). The tissue was incubated at 4°C over night and then removed from RNA*later* and stored at *−*70°C until RNA extraction as described below. The experiment was carried out in strict accordance with the recommendations in the Guide for the Care and Use of Laboratory Animals of the National Institutes of Health and all procedures were approved by the Johns Hopkins University Animal Care and Use Committee.

### RNA Extraction and Real Time PCR

To extract RNA from cultures, bacterial pellets were suspended in 1 ml Trizol and transferred to a screw-cap tube with ∼1 g 0,1 mm zirconia beads (Research Products International Corp. Mount Prospect, IL) followed by 9×30-second bead-beating cycles at 2800 oscillations/minute with a 1 minute rest on ice between each cycle. Cellular debris was pelleted by a 5 minute centrifugation at 13000 rpm at 4°C and cleared lysate transferred to a new tube. The samples were incubated 2–3 minutes at room temperature followed by the addition of 200 µl isopropanol, 15s vigorous shaking, and another 2–3 minute incubation before a 15 minute centrifugation at 12.000×g, 4°C. The aqueous phase was carefully transferred to a new tube and mixed with an equal volume of 100% ethanol. The samples were then applied to RNeasy spin columns (Qiagen, Valencia, CA) and further processed according to manufacturer’s instructions. Lung homogenate and J774 cell samples were briefly bead-beaten with 1 mm glass beads (VWR, Bridgeport, NJ) in Trizol to disrupt the mammalian cell membranes, dissolving the murine RNA while leaving intact *M.tb* cells, which were collected in the pellets after a 5 minute centrifugation at 13000 rpm. The eukaryotic RNA-containing supernatant was removed, and the bacterial pellets were resuspended in freshTrizol and processed as above. The RNA was DNase treated with TURBO DNase (Ambion, Life Technologies, Grand Island, NY) and reverse-transcribed using the iScript cDNA synthesis kit (BioRad, Hercules, CA) according to the manufacturer’s instructions.

Real time PCR was performed with iQ SYBR Green on a MyiQ cycler (BioRad, Hercules, CA) in 25 µl reactions. Primers for *whiB1*-7 and housekeeping gene *sigA* are described in [12]. A no reverse transcriptase control on input RNA was included for each sample. All experiments were done in biological and technical triplicates. CT values were normalized against *sigA* expression, and fold change was calculated by the −2^ΔΔCt^ method.

### Statistical Analysis

1-tailed Student’s t-tests assuming equal variance were performed on treated vs. untreated samples after *sigA* normalization. p-values are indicated with asterisks in figures as follows: * = p<0.05, ** = p<0.01, *** = p<0.001. All data are presented as mean ± standard deviation.

### Overexpression of *whiB 1-7* in *M.tb*

*whiB1-7* were cloned introducing NdeI and PacI sites and ligated in front of the *ace* promoter in the plasmid pSCW35 [22], [23]. Plasmids were electroporated into *M.tb* CDC1551 and selected for on kanamycin-containing plates. Transformants were verified to carry the plasmid and expanded in 7H9 containing kanamycin. Acetamide was added to a final concentration of 0.2% to OD_600_ 0.5 cultures in triplicate. Bacteria were harvested after 24 h of induction. Acetamide treated bacteria carrying the empty vector plasmid pSCW38 was used as negative control for the fold change calculations.

## Supporting Information

Figure S1 **Measuring oxygen in the oxygen depletion experiment.** Setup of the OXY MICRO device with computer, syringe-based oxygen microsensor and temperature probe for temperature compensation (left panel). The right panel shows a close-up of a sample being measured with the temperature probe (thick metal rod to the right) and the thin fiber-optic glass microsensor extended from its needle (left). The excitation light by which oxygen is measured is seen at the tip of the microsensor. Samples are aspired from septated culture tubes using a syringe with thin needle and carefully transferred to test tube to minimize air exposure. Oxygen level is read when temperature is stabilized.
(TIF)
