# Supplementary figures and images for: Gene Expression of Mycobacterium tuberculosis Putative Transcription Factors whiB1-7 in Redox Environments
Source: PLoS One. 2012 Jul 19;7(7):e37516. doi: 10.1371/journal.pone.0037516 (PMC3400605; doi:10.1371/journal.pone.0037516)

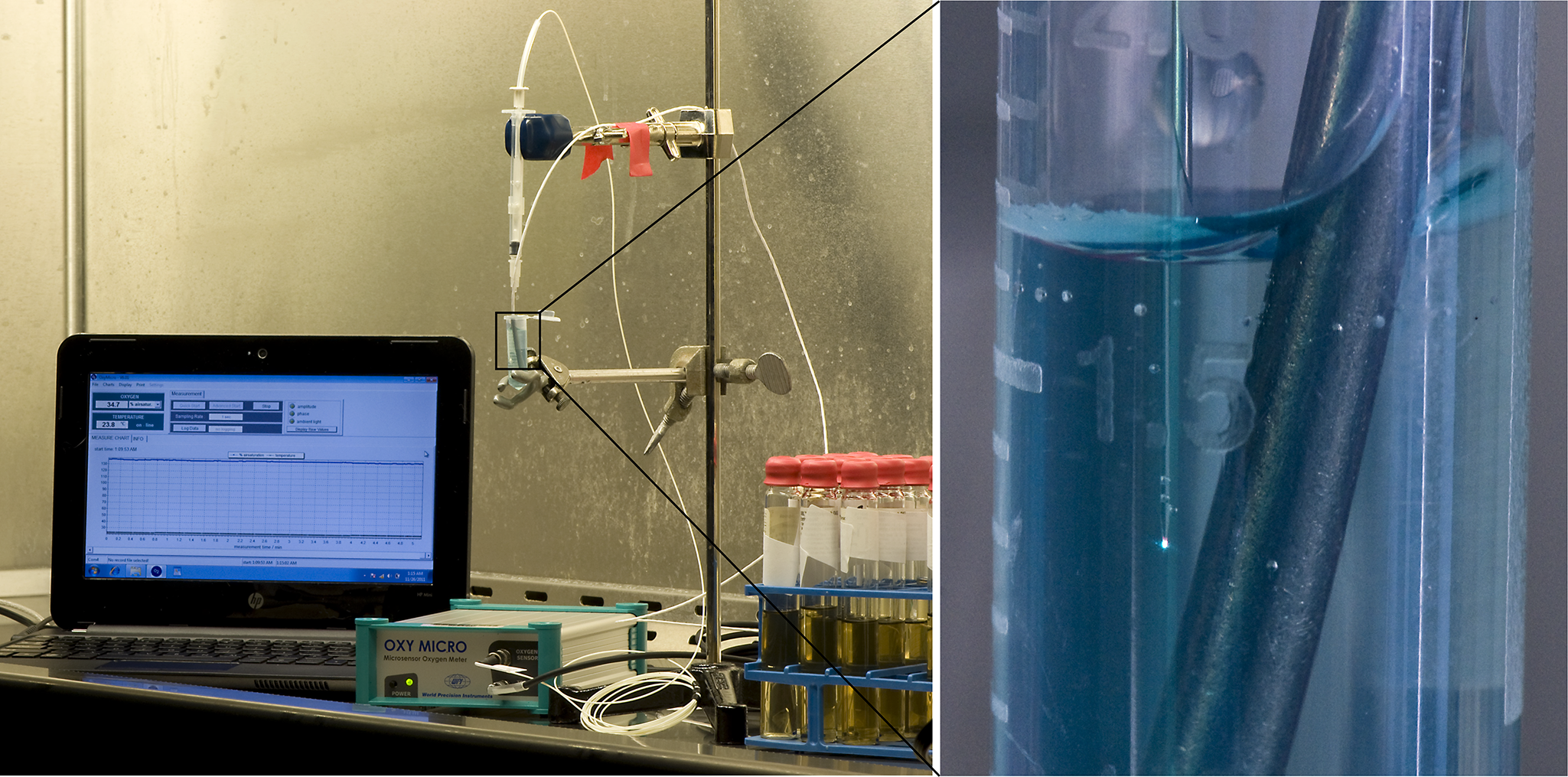

Supplement: Figure S1 — Measuring oxygen in the oxygen depletion experiment. Setup of the OXY MICRO device with computer, syringe-based oxygen microsensor and temperature probe for temperature compensation (left panel). The right panel shows a close-up of a sample being measured with the temperature probe (thick metal rod to the right) and the thin fiber-optic glass microsensor extended from its needle (left). The excitation light by which oxygen is measured is seen at the tip of the microsensor. Samples are aspired from septated culture tubes using a syringe with thin needle and carefully transferred to test tube to minimize air exposure. Oxygen level is read when temperature is stabilized. (TIF) [file pone.0037516.s001.tif]
